# Indications for Operation in Disease of the Internal Organs

**Published:** 1907-03

**Authors:** 


**Indications for Operation in Disease of the Internal Organs.** By Professor HERMANN SCHLESINGER, M.D. Authorised English Translation by KEITH W. MONSARRAT, M.B., F.R.C.S. Pp. xv., 498. Bristol: John Wright and Co. 1906.

This book is written essentially for the practitioner, in order to enable those who are not in hospital practice to arrive at an independent opinion on the advisability of operation in cases of internal lesion. In each chapter there are remarks on etiology, pathological anatomy, clinical course, diagnosis, and differential diagnosis, to give a general grasp of the condition under consideration, and a small selection from the literature is also added at the end of each chapter.

Being written by a physician, it is the more valuable from the surgical point of view; for though the writer is clearly in sympathy with surgical methods under the circumstances defined in the various chapters, he is nevertheless guided by a clear judgment in recommending operation only in such conditions as have failed to respond to medical treatment, or are clearly unsuitable for it. The statements are concise, and the end in view is clearly and definitely stated. We consider it a most useful book for those in general practice who are often in doubt as to the necessity of calling in a surgeon.

A word of praise and thanks must also be accorded to the translator, who has so ably put into English a work which it is highly desirable should be possessed by many who have not a sufficient knowledge of German to read the original.

